# *De Novo* Sequencing and Transcriptome Analysis of the Central Nervous System of Mollusc *Lymnaea stagnalis* by Deep RNA Sequencing

**DOI:** 10.1371/journal.pone.0042546

**Published:** 2012-08-01

**Authors:** Hisayo Sadamoto, Hironobu Takahashi, Taketo Okada, Hiromichi Kenmoku, Masao Toyota, Yoshinori Asakawa

**Affiliations:** 1 Faculty of Pharmaceutical Sciences at Kagawa Campus, Tokushima Bunri University, Shido, Sanuki-City, Kagawa, Japan; 2 Institute of Pharmacognosy, Tokushima Bunri University, Yamashiro-cho, Tokushima-City, Japan; Tokai University, Japan

## Abstract

The pond snail *Lymnaea stagnalis* is among several mollusc species that have been well investigated due to the simplicity of their nervous systems and large identifiable neurons. Nonetheless, despite the continued attention given to the physiological characteristics of its nervous system, the genetic information of the *Lymnaea* central nervous system (CNS) has not yet been fully explored. The absence of genetic information is a large disadvantage for transcriptome sequencing because it makes transcriptome assembly difficult. We here performed transcriptome sequencing for *Lymnaea* CNS using an Illumina Genome Analyzer IIx platform and obtained 81.9 M of 100 base pair (bp) single end reads. For *de novo* assembly, five programs were used: ABySS, Velvet, OASES, Trinity and Rnnotator. Based on a comparison of the assemblies, we chose the Rnnotator dataset for the following blast searches and gene ontology analyses. The present dataset, 116,355 contigs of *Lymnaea* transcriptome shotgun assembly (TSA), contained longer sequences and was much larger compared to the previously reported *Lymnaea* expression sequence tag (EST) established by classical Sanger sequencing. The TSA sequences were subjected to blast analyses against several protein databases and *Aplysia* EST data. The results demonstrated that about 20,000 sequences had significant similarity to the reported sequences using a cutoff value of 1e-6, and showed the lack of molluscan sequences in the public databases. The richness of the present TSA data allowed us to identify a large number of new transcripts in *Lymnaea* and molluscan species.

## Introduction

The pond snail *Lymnaea stagnalis* has large identifiable neurons and a simple central nervous system (CNS). Many researchers have therefore used this animal model to investigate the cellular and molecular mechanisms related to various behaviors, such as respiration, feeding, learning and memory [1]–[3]. Despite the continued attention given to the physiological characteristics of the identified neurons, the genetic information of *Lymnaea* has not yet been fully explored. Thus, for molecular biological investigations, researchers have made numerous efforts to identify new genes before studying their function [4]–[6]. Previously, two *Lymnaea* expression sequence tag (EST) databases were established by classical Sanger sequencing [7], [8]. Nevertheless, they are still insufficient to perform transcriptome analysis and improved transcriptome data is continuously needed.

A recently developed technology, deep RNA sequencing (RNA-seq), produces at least 100 to 1,000 times higher throughput than classical Sanger sequencing [9]. Commercially available RNA-seq technologies, such as the Illumina Genome Analyzer and Roche Genome Sequencer FLX system (GS FLX), are now widely applied for RNA and genome sequencing, and the features of several RNA-seq platforms have been well defined [10], [11]. Simply put, GS FLX provides longer reads and has been reported to enable more efficient assembly compared to the Illumina sequencer. By contrast, the Illumina sequencer was reported to produce large amounts of data with short reads at a lower cost. More recent studies have reported that longer read data (>75 bp) can be obtained using an Illumina sequencer and the developed assembly programs enable researchers to perform *de novo* transcriptome sequencing at a low cost [12], [13]. In this study, we examined the RNA-seq method and several *de novo* assembly programs for their ability to provide transcriptome data of *Lymnaea* CNS using an Illumina sequencer.

**Figure 1:**
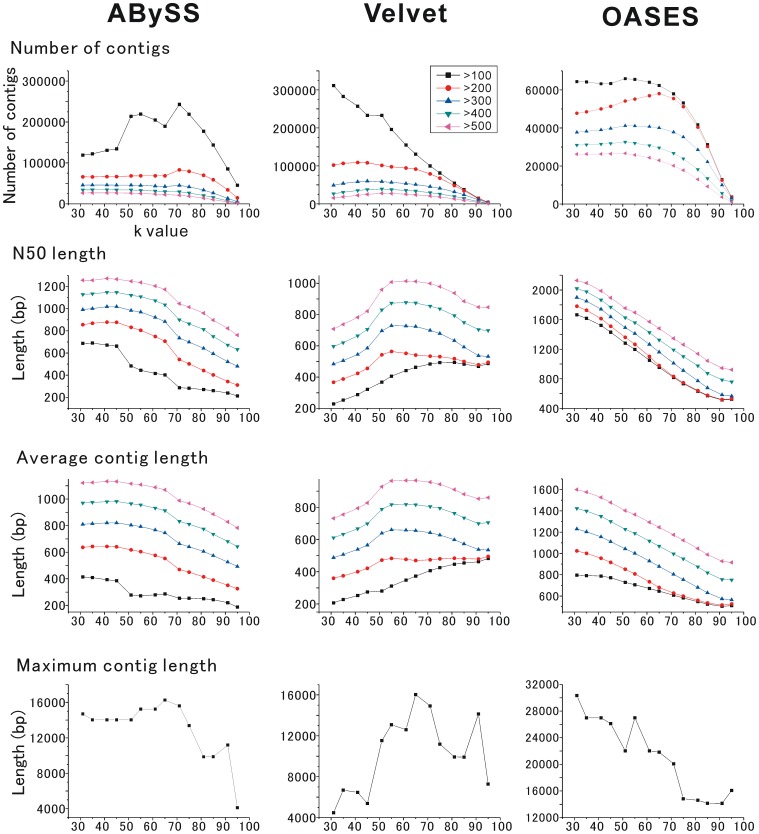
Comparison of *de novo* assembly with a distinct k-value. Three assembly programs, ABySS, Velvet and OASES, were tested with a distinct k-mer from 31 to 95. In each assembly program, the number of contigs, the N50 length, and the average and maximum contig length were calculated using the assembled contigs longer than 100 bp (black), 200 bp (orange), 300 bp (blue), 400 bp (green) and 500 bp (pink).

## Materials and Methods

### Snails

**Figure 2:**
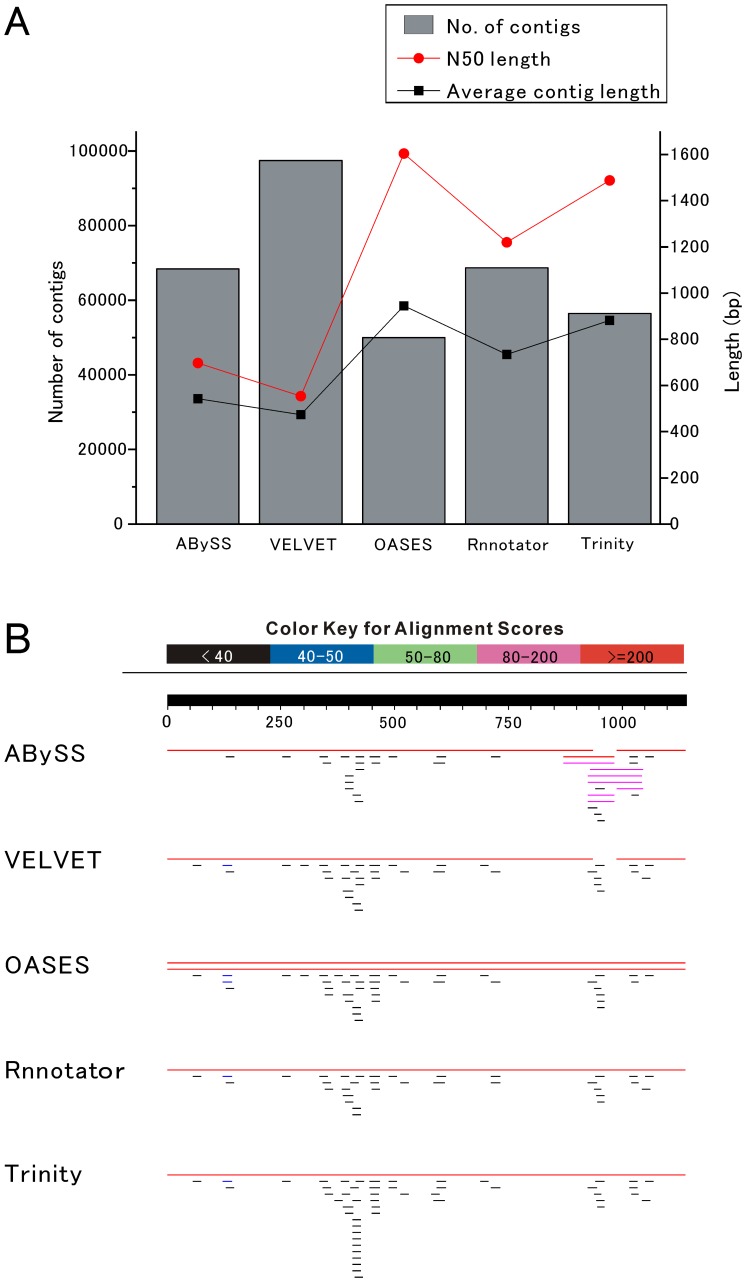
Comparison of *de novo* assembly quality among the different programs. (A) Overall comparison of the results from five assembly programs. The bars indicate the number of contigs longer than 200 bp (left axis). The red lines indicate the N50 length and the black lines indicate average contig length in bp (right axis). (B) Blast requests of LymCREB2 CDS (1,140 bp) for differently assembled contigs. The black line above represents the query sequence and the colored lines below represent the results of the similarity of hit contigs in the databases. The alignment scores are indicated by five colors in the label at the top.

Specimens of *Lymnaea stagnalis* with a 25 mm shell were maintained in tap water and fed on Komatsuna, Japanese mustard spinach, on a 12∶12 light-dark cycle at 20°C. Snails were anesthetized by 25% Listerine® before dissection. For RNA extraction, the isolated CNSs were frozen in liquid nitrogen.

### RNA Extraction and Transcriptome Sequencing

The *Lymnaea* CNSs were dissected and immediately frozen in liquid nitrogen. RNA was extracted from 80 dissected CNSs using a FastPure RNA kit (Takara Bio, Shiga, Japan) and treated with DNase I (Takara). The quality and quantity were assessed on a 2100 Bioanalyzer using an RNA 6000 Nano kit (Agilent Technologies, Palo Alto, CA). After the measurement of RNA concentration, equal volumes of total RNA from each group were pooled and used for library preparation. Libraries were prepared using a TruSeq™ RNA sample preparation kit with a Low-Throughput protocol (Illumina Inc., San Diego, CA) according to the manufacturer’s instructions. The DNA concentration of the cDNA library was measured using a 2100 Bioanalyzer using a DNA 1000 kit (Agilent Technologies) and diluted to 4 nM, and a 120 µl aliquot was used to generate clusters on a Single-Read flow cell using the cBOT (Illumina) and sequenced on the GAIIx using the SBS 36-cycle Sequencing Kit v5. One lane for each group was sequenced as 100-bp reads, and image analysis and base calling were performed with SCS2.8/RTA1.8 (Illumina). FASTQ file generation and the removal of failed reads were performed by CASAVA ver.1.8.2 (Illumina).

**Table 1:** The *Lymnaea* TSA obtained from Rnnotator assembly.

Total number of reads	81,851,004
Number of used reads	20,020,897
Number of unused reads	61,830,107
Number of non-redundant transcripts	116,355
Number of transcript isoforms	4,222
Total size of transcriptome (bp)	76,435,832
N50 length (bp)	1,438
Average contig length (bp)	656
Largest contig length (bp)	26,147
Average coverage (rpkm)	8.594

### Application of Published *de novo* Methods

Reads obtained using an Illumina sequencer were *de novo* assembled using ABySS ver. 1.3.2 [14], Velvet ver. 1.1.07 [15], OASES ver. 0.2.01 [16], Trinity 2011-08-20 [17], [18] or Rnnotator ver. 2.4.12 [19]. ABySS and Velvet were run using different k-mer lengths of 31 to 95 along with other default parameters. OASES was run using k-mers of 31 to 95 followed by merging the results by running the first stage of Velvet analysis. Trinity and Rnnotator were run with the default parameters.

**Figure 3:**
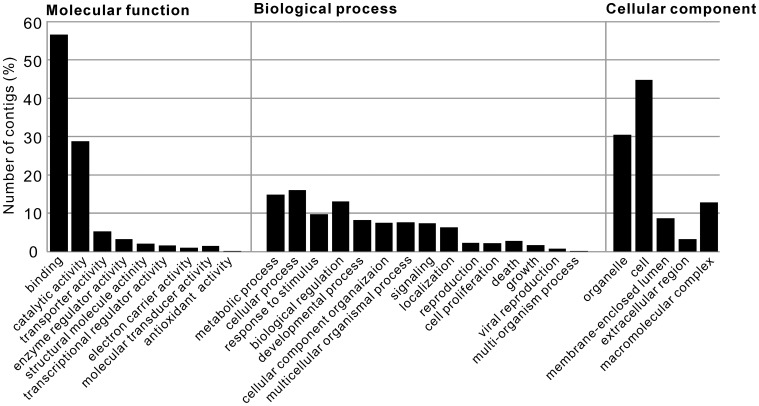
Gene ontology distribution for the *Lymnaea* TSA. Gene ontology distribution of the *Lymnaea* TSA derived from BLAST2GO. The results are summarized as molecular functions, biological processes and cellular components. The x-axis represents the percentage of contigs divided by the total number of cells counted with the given level 2 GO terms.

### BLAST Annotation

With the dataset of 82,127 contigs (>200 bp) assembled by Rnnotator, the BLAST program [20] was performed for the blastx homology search among the databases: Swiss Protein (Swissprot Release_12_2011), the NCBI Protein Reference sequences (Refseq, Release_14_01_2012) and the Invertebrate Protein Reference sequences (Invertebrate Refseq, Relase50_ 08_11_2011), respectively. For similarity searches of the molluscan ESTs, the available *Lymnaea stagnalis* and *Aplysia californica* EST sequences were downloaded from GenBank [8], [21]. The present contigs identified by Rnnotator (116,355 contigs, >100 bp) and the previous *Lymnaea* EST dataset (11,697 sequences) were used for blastn search bidirectionally with an e-value cutoff of 1e-100. For comparison with *Aplysia* ESTs, the contigs identified by Rnnotator (116,355 contigs, >100 bp) were used to perform blastn and tblastx homology searches in the *Aplysia* EST database (255,605 sequences) with an e-value cutoff of 1e-6.

**Table 2:** Blast hits of the *Lymnaea* TSA to different protein databases.

e-value	Number of contigs havinga blast hit in Swissprot	(%)	Number of contigs having ablast hit in Refseq	(%)	Number of contigs having a blasthit in Invertebrate Refseq	(%)
0	1,261	1.5	1,510	1.8	1,430	1.7
1.00E-150	1,799	2.2	2,120	2.6	2,018	2.5
1.00E-100	3,344	4.1	3,925	4.8	3,767	4.6
1.00E-50	6,670	8.1	7,685	9.4	7,510	9.1
1.00E-20	11,314	13.8	13,202	16.1	12,957	15.8
1.00E-10	14,474	17.6	17,523	21.3	17,318	21.1
1.00E-06	16,534	20.1	20,642	25.1	20,578	25.1
total	82,127		82,127		82,127	

### Gene Ontology Annotation

Using the 82,127 sequences of Rnnotator assembled contigs (>200 bp), assignment of gene ontology (GO) terms was performed by importing the GO-numbers obtained by a blastx search against the Swissprot database into BLAST2GO (version 2.5.0; http://www.blast2go.org/) [22]. Categorization of the BLAST matches and construction of column bar graphs was conducted using the standard graph configurations including level two GO-terms only.

**Figure 4:**
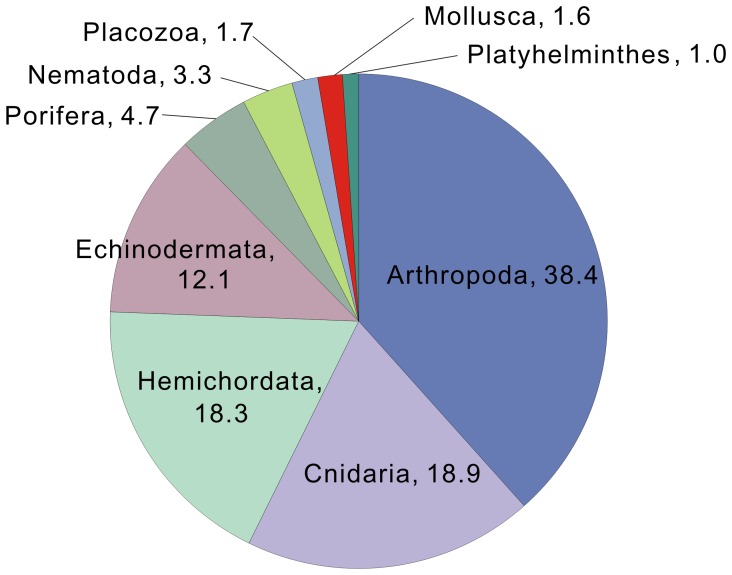
Phylum distributions of top blast hit species in the invertebrate protein database. The percentages of phylum for top blast hit species are shown for the results with a cutoff e-value of 1e-10.

### The Phylogenetic Analyses of Newly Identified Proteins

The computer program MEGA4 [23] was used to construct a neighbor-joining phylogeny from Poisson amino acid distances. Bootstrap values are given for each branch. The accession numbers for the sequences of tyrosine hydroxylase included in the tree are as follows: *Mus musculus*: NP_033403; *Homo sapiens*: AAI04968; *Bos taurus*: NP_776309; *Gallus gallus*: NP_990136; *Xenopus laevis*: NP_001091392; *Danio rerio*: NP_571224; *Branchiostoma floridae*: AAZ32939; *Strongylocentrotus purpuratus*: XP_786206; *Pediculus humanus corporis*: XP_002430839; *Apis mellifera*: NP_001011633; *Bombus impatiens*: XP_003486645; *Tribolium castaneum*: NP_001092299; *Bombyx mori*: ADV56714; *Manduca sexta*: ABQ95973; *Caenorhabditis elegans*: P90986. The accession numbers for the sequences of dopa decarboxylase included in the tree are as follows: *Homo sapiens*: NP_000781; *Bos taurus*: NP_776332; *Mus musculus*: NP_057881; *Monodelphis dome*: XP_001379620; *Anolis carolinensis*: XP_003222345; *Meleagris gallopavo*: XP_003204897; *Gallus gallus*: XP_419032; *Xenopus laevis*: NP_001104211; *Danio rerio*: NP_998507; *Pediculus humanu*s *corporis*: XP_002426339; *Bombus terrestrius*: XP_003399661; *Bombyx mori*: NP_001037174; *Aedes aegypti*: XP_001648263; *Apis mellifera*: XP_394115; *Drosophila melanogaster*: P05031; *Caenorhabditis elegans* tyrosine decarboxylase: NP_495744. The accession numbers for the sequences of dopamine beta-hydroxylase included in the tree are as follows: *Bos taurus*: NP_851338; *Homo sapiens*: NP_000778; *Mus musculus*: NP_620392; *Ornithorhynchus anatinus*: XP_001505587; *Gallus gallus*: XP_415429; *Xenopus tropicalis*: XP_002942324; *Danio rerio*: NP_001103164; *Dugesia japonica*: BAG86630; *Aedes aegypti*: XP_001661114; *Drosophila melanogaster*: NP_788884; *Bombyx mori*: NP_001243923; *Acyrthosiphon pisum*: XP_001951145; *Pediculus humanus corporis*: XP_002425605; *Tribolium castaneum*: XP_974169; *Nasonia vitripennis*: XP_001602880; *Apis mellifera*: NP_001071292; *Caenorhabditis elegans*: Q9XTQ6.

**Table 3:** Comparison of the present *Lymnaea* TSA and the previously reported *Lymnaea* EST.

e-value	Number of the previous Lymnaea ESThaving a blast hit in the present TSA	(%)	Number of the present Lymnaea TSA having ablast hit in the previous EST	(%)
0	8,398	71.8	4,520	3.9
1.00E-150	8,783	75.1	4,936	4.2
1.00E-100	9,388	80.3	6,049	5.2
total	11,697		116,355	

## Results

### *Lymnaea* CNS Transcriptome Assembly

The messenger RNA samples were prepared from pooled *Lymnaea* CNSs of 80 animals, and sequencing was performed in two lanes of a flow cell using an Illumina Genome Analyzer IIx. To obtain better assembly for *Lymnaea* CNS transcriptome, we here examined five *de novo* assembly tools, ABySS [14], Velvet [15], OASES [16], Trinity [17], [18] and Rnnotator [19].

**Table 4:** Comparison of the *Lymnaea* TSAs and *Aplysia* EST.

e-value	Number of contigs having a blastn hitin Aplysia EST	(%)	Number of contigs having a tblastx hitin Aplysia EST	(%)
0	11	0.0	13	0.0
1.00E-150	24	0.0	245	0.2
1.00E-100	124	0.1	2,050	1.8
1.00E-50	768	0.7	5,669	4.9
1.00E-20	2,262	1.9	10,314	8.9
1.00E-10	3,733	3.2	14,437	12.4
1.00E-06	4,806	4.1	18,275	15.7
total	116,355		116,355	

First, to choose the best k-mer length for *de novo* assembly, we applied different k-mer lengths for ABySS, Velvet and OASES. We first used a dataset from one lane with 41 M reads to save the memory usage. The results with distinct k-mer values were compared using the performance criteria, contig number, N50 length, average contig length and maximum contig length. The N50 length was calculated by adding the lengths of contigs from long to short until the summed length exceeded 50% of the total length of all contigs. It is generally accepted that larger values of these criteria indicate better assembly performance. The result showed that the values were extensively varied according to the length of k-mer in each assembly (Fig. 1), and the data of contigs longer than 200 bp were chosen to assess the assembly quality. This was because the results of the three programs consistently revealed that the data including short contigs (>100 bp) yielded less accurate results than the data with longer contigs. For example, the trial using the program ABySS (Fig. 1, left column) revealed that the criteria values obtained using short contigs (>100 bp, black lines) largely differed from those obtained using long contigs (>200 bp, colored lines).

**Figure 5:**
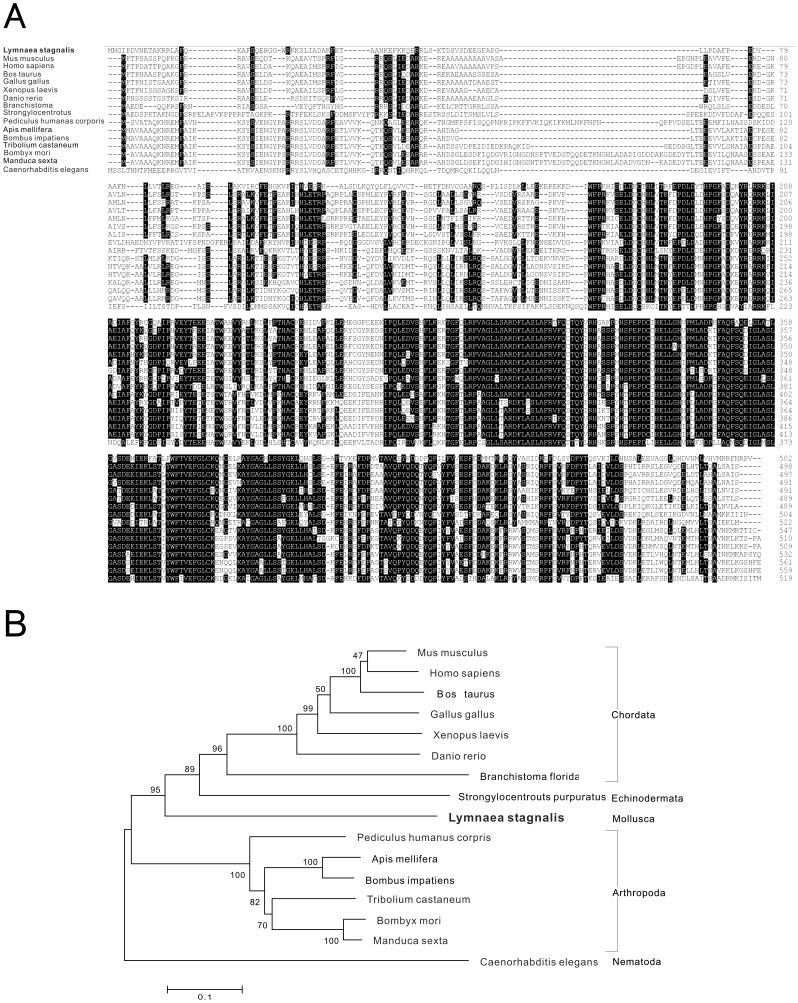
Protein sequence alignment and phylogenetic tree of tyrosine hydroxylase. Protein sequence alignment of tyrosine hydroxylase (A) and phylogenetic analyses (B) were conducted by the neighbor-joining method using the MEGA4 program with the *C. elegans* tyrosine hydroxylase as outgroup. The percentage of replicate trees in which the associated taxa clustered together in the bootstrap test is shown next to the branches. The scale bars indicate the estimated evolutionary distance in the units of the number of amino acid substitutions per site.

In the result of ABySS assembly, with a k-mer length of 65, the criteria values of the number of contigs, N50 length and average contig length seemed to plateau, and the maximum contig length was longest. Velvet assembly produced the best values of N50 length, average contig length and maximum contig length, with a k-mer length of 55. In the case of OASES, the data included a large number of variants, and we used the dataset with the longest variants. The results clearly demonstrated that the criteria values were better with a shorter length of k-mer for OASES, and seemed to plateau with a k-mer length of 31. According to the obtained results, the best k-mer lengths were determined as 65 for ABySS, 55 for Velvet and 31 for OASES, respectively.

**Figure 6:**
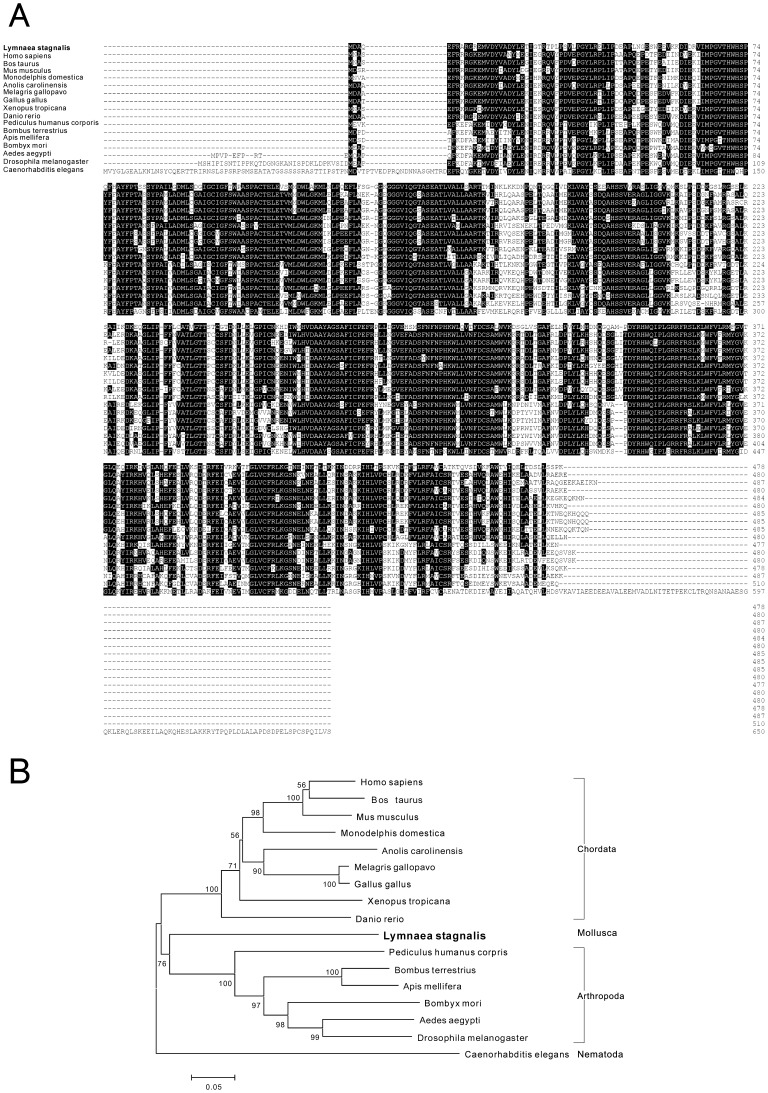
Protein sequence alignment and phylogenetic tree of dopa decarboxylase. Protein sequence alignment of dopa decarboxylase (A) and phylogenetic analyses (B) were conducted by the neighbor-joining method using the MEGA4 program with the *C. elegans* tyrosine decarboxylase as outgroup. The percentage of replicate trees in which the associated taxa clustered together in the bootstrap test is shown next to the branches. The scale bars indicate the estimated evolutionary distance in the units of the number of amino acid substitutions per site.

We then performed assemblies using each of the five programs and compared the results using the contigs longer than 200 bp (Fig. 2A). Because splicing variants were included in the results of the OASES, Trinity and Rnnotator assemblies, the longest variant for a singular transcript was extracted and used as the data for the comparison. As a result, the assemblies of the OASES, Trinity and Rnnotator programs appeared to be better than those of ABySS and Velvet. The N50 lengths in the ABySS and Velvet data were obviously shorter than those yielded by the programs OASES, Trinity and Rnnotator (707 for ABySS; 564 for Velvet; 1,614 for OASES; 1,230 for Rnnotator; 1,497 for Trinity) and the values of average contig length showed a similar tendency (553 for ABySS; 483 for Velvet; 955 for OASES; 744 for Rnnotator; 892 for Trinity). In contrast, Velvet produced the largest number of contigs among the programs, and ABySS also produced a larger contig number than OASES and Trinity (68,378 for ABySS; 97,492 for Velvet; 50,021 for OASES; 68,734 for Rnnotator; 56,446 for Trinity). So far, these results indicated that ABySS and Velvet produced much greater numbers of shorter contigs than OASES, Trinity and Rnnotator.

**Figure 7:**
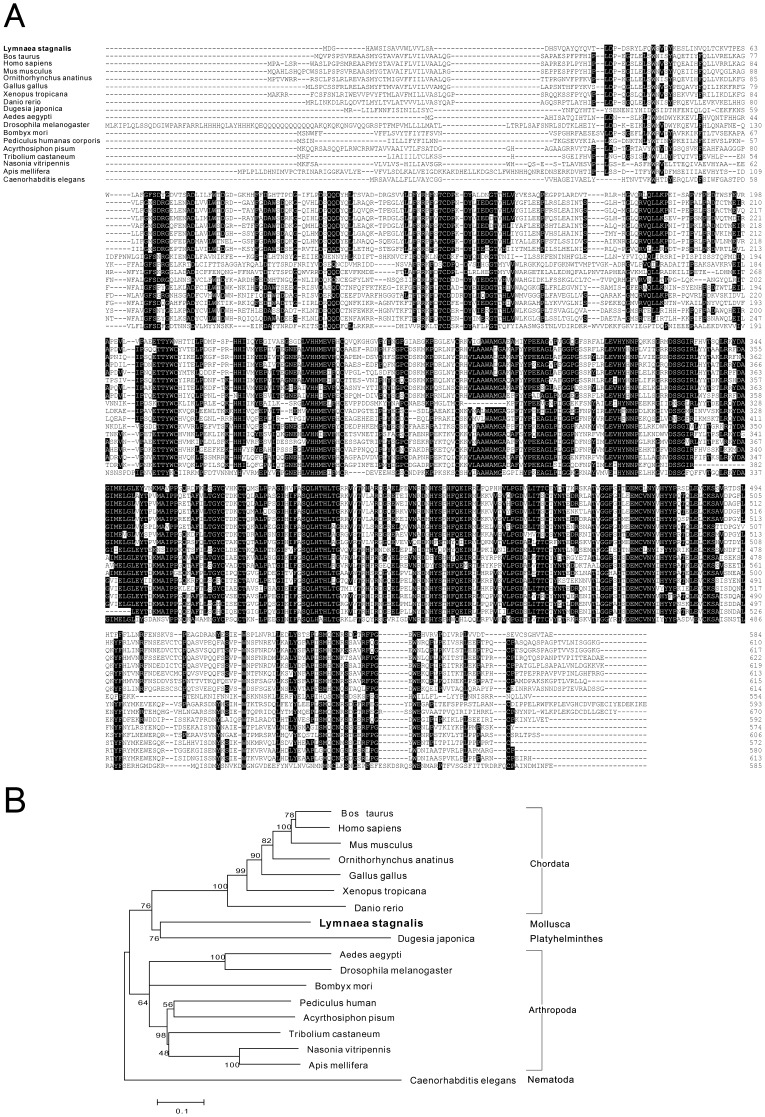
Protein sequence alignment and phylogenetic tree of tyramine beta hydroxylase. Protein sequence alignment of tyramine beta hydroxylase (A) and phylogenetic analyses (B) were conducted by the neighbor-joining method using the MEGA4 program with the *C. elegans* of tyramine beta hydroxylase as outgroup. The percentage of replicate trees in which the associated taxa clustered together in the bootstrap test is shown next to the branches. The scale bars indicate the estimated evolutionary distance in the units of the number of amino acid substitutions per site.

On the other hand, a previous report showed that the values of the contig number, N50 length and average contig length are not in themselves sufficient to assess the accuracy of assembly [24]. Therefore, BLAST methodology was additionally examined to check each contig length and the coverage of known sequences (Fig. 2B). We here applied a single open reading frame (ORF) of LymCREB2 [2], and observed that it was covered with a single contig in the datasets of OASES, Trinity and Rnnotator. By contrast, it was covered with 3 and 2 shorter contigs in the datasets of ABySS and Velvet, respectively. The sequence coverage with a known sequence also indicated that the ABySS and Velvet programs were inferior to OASES, Trinity and Rnnotator in assembly performance.

Among the programs OASES, Trinity and Rnnotator, the results so far do not show a consistent advantage or any significant superiority for any particular program. The contig number was the largest in the dataset of Rnnotator and the N50 lengths and average lengths of contigs were longer in the datasets of OASES and Trinity. However, it should be noted that the datasets of OASES, Trinity and Rnnotator include plural variants for a singular transcript and the numbers of variants in OASES and Trinity datasets were much larger than that of Rnnotator dataset (OASES: 54,601; Trinity: 44,062; Rnnotator: 2,675). For the following gene ontology (GO) analysis, a dataset including redundant sequences is not suitable. Therefore, the Rnnotator program was selected for assembly of the full dataset.

The dataset from two lanes of a flow cell (81.9 M reads) was then assembled with Rnnotator and the results are summarized in Table 1. The redundant read and low quality sequences were removed at the preprocessing step, and 20 M reads were used for assembly. As a result, we obtained 116,355 non-redundant sequences as a *Lymnaea* transcriptome shotgun assembly (TSA) and deposited the TSA data in DDBJ (the DNA Data Bank of Japan, accession number FX180119 to FX296473). The data had a total size of about 76.4 Mbp, N50 length of 1,438 bp, and average contig length of 656 bp. We further confirmed that the longest contig (26,147 bp, accession number: FX180119), coding a homologue of titin protein, had a large open reading frame of 25,191 bp without any frame shift or stop codon (data not shown), indicating that Rnnotator successfully assembled the present sequence data. Rnnotator assembly also produced the value of reads per kilo per million (rpkm) as an index of the expression level [19]. The present data showed a maximum rpkm value of 64707.57 and an average rpkm value of 8.59.

### Gene Ontology Mapping

We then classified the *Lymnaea* TSA sequences associated with GO terms at the protein level annotation using BLAST2GO [25]. The 82,127 contigs longer than 200 bp were subjected to blastx analysis against the Swiss Protein (Swissprot) database using a cutoff e-value of 1e-6. As a result, 16,534 sequences (20.1%) had significant matches in the Swissprot database, and these were functionally annotated with GO terms by BLAST2GO. A domain analysis was also performed with InterProScan, and 1,454 sequences without balstx hits were further combined. Then, 16,002 sequences with annotated GO terms were remapped on a reduced set of GO categories (GO-slim) and classified into three categories: molecular function, biological process and cellular component. Figure 3 is the summary of the classification of annotated *Lymnaea* TSA sequences at level 2. In the molecular function classification, the clusters relating to “binding” and “catalytic activity” were enriched (56.7% and 28.8%, respectively). In the biological process classification, no cluster was specifically enriched. For the cellular component classification, the cluster sizes of “cell” (30.5%) and “organelle” (44.8%) were relatively large compared to those of “membrane-enclosed lumen,” “extracellular region” and “macromolecular complex.”

### Blast Search in Public Protein Databases

Next, the *Lymnaea* TSA (>200 bp) was subjected to blastx analysis against three different databases, Swissprot, NCBI Protein Reference sequences (Refseq) and Invertebrate Protein Reference sequences (Invertebrate Refseq). The results with several cutoff values are summarized in Table 2. Interestingly, the results were not largely varied between blast analyses against invertebrate protein and universal protein databases. For instance, using cutoff values of 1e-6, 16,534 contigs (20.1%) of *Lymnaea* TSA sequences showed significant hits with an existing entry in Swissprot, and 20,642 and 20,578 contigs had significant hits to the Refseq and Invertebrate Refseq databases, respectively. With a strict cutoff value of 0, the numbers of contigs having blast hits was not largely varied (1,261 for the Swissprot; 1,510 for the Refseq; 1,430 for the Invertebrate Refseq database).

We further compared the phylum distribution of top hit species in a blastx search and observed that the percentages of mollusca phylum were clearly lower than those of the other phyla. Using a cutoff value of 1e-10 for the blastx analysis against Invertebrate Refseq, 38.4% of total hit sequences had similarity to arthropoda sequences (Fig. 4). On the other hand, only 1.6% had blast hits to mollusca sequences. Moreover, the phylum distributions did not vary among the results when more stringent cutoff values of 1e-50 and 1e-150 were used (43.6% and 40.9% for arthropoda; 2.8% and 5.5% for mollusca, respectively). These results demonstrated that the number of molluscan sequences in the public databases is not sufficient to analyze the transcriptome data.

### Comparison with the Previously Reported *Lymnaea* EST Sequences

To compare the present TSA data with previously reported *Lymnaea* EST sequences, we next performed a bidirectional blastn analysis (Table 3). In GenBank, 11,697 EST from *Lymnaea* CNS [8] are registered. They include sequences shorter than 100 bp, and thus we can use the whole dataset including short contigs (116,355 contigs, >100 bp) in this analysis. As a result, over 80% of the previous EST data had blast hits to the present TSA data using a stringent cutoff value of 1e-100. By contrast, only 5% of the present TSA sequences had hits to the previous EST data with the same cutoff value. The results clearly demonstrated that about 95% of the present TSA, 110,000 sequences, were the newly identified transcripts.

We further noted that the numbers of hit sequences differed according to the direction of the blast search. Using a strict cutoff value of 0, 8,398 sequences of the previous EST and 4,520 sequences of the present TSA showed blast hits against the data of the other. Closer inspection revealed that multiple sequences in the previous EST showed blast hits to a single contig in the present TSA. In addition, the average lengths of these 8,398 EST sequences were approximately three times shorter than those of the 4,520 TSA sequences (the EST, 823 bp; the TSA, 2,487 bp). The results indicated that multiple short sequences of the previous EST had similarity to different parts of a single long contig in the present TSA. On the other hand, 4,520 contigs of the present TSA had blast hits to independent sequences of the previous EST. These results clearly indicated that the present RNA-seq analysis largely improved the coverage and the contig length of transcriptome data from *Lymnaea*.

### Comparison with *Aplysia* EST Sequences

To assess transcriptome coverage of the present data, we further performed a blastn analysis against *Aplysia californica* EST (255,605 sequences), which is the largest molluscan EST available in the public databases [21]. As a result, with a cutoff value of 1e-6, only 4,806 sequences (4.1%) of *Lymnaea* TSA showed blast hits (Table 4). A tblastx analysis was also performed to compare the translated sequences of TSA. Using a lenient cutoff value of 1e-6, 18,275 sequences (15.7%) of *Lymnaea* TSA data showed tblastx hits to the *Aplysia* EST data.

The tblastx result was further compared with the result of blast analysis against Invertebrate Refseq (Table 2). Using the same dataset (>200 bp) and a stringent cutoff value of 0, 1,430 contigs showed blast hits to Invertebrate Refseq, while only 13 contigs did so to *Aplysia* EST. The number of contigs showing similarity to *Aplysia* EST was clearly less than that showing similarity to sequences of other phylum species. The results revealed that the present *Lymnaea* TSA data were not comparable to the present *Aplysia* EST data.

### Newly Identified Transcripts in Molluscan Species

Finally, to verify the accuracy of assembly, we observed sequences of particular contigs with a single complete open reading frame (ORF) as representative examples. According to the annotated result, we here isolated the sequences of monoamine synthesis enzymes that have not yet been identified in molluscan species. Because monoamines (e.g. dopamine, octopamine and serotonin) play critical functions in neuroregulatory mechanisms in animal species, the genes of monoamine synthesis enzymes should be expressed in molluscan nervous system.

First, a homolog sequence of *Lymnaea* tyrosine hydroxylase (TH) was isolated according to the blast search result. The enzyme TH catalyzes the conversion of L-tyrosine to L-dihydroxyphenylalanine (L-DOPA), the rate-limiting step in the dopamine synthesis pathway. The *Lymnaea* TH sequence (DDBJ accession number: FX186872) had a length of 2,331 bp and encoded 502 amino acids. Amino acid alignment was performed with hit sequences identified by blastx searches in public databases, including the Swissprot, GenBank and Refseq protein databases. Using the ClustalW program in the MEGA4 software package [23], multiple alignments were performed and the result revealed amino acid sequences of TH were highly conserved among animal species (Fig. 5A). In addition, a phylogenetic tree, constructed by the neighbor-joining method, showed that *Lymnaea* TH was distant from the other phylum species THs, and forms a deep phylogenetic branch (Fig. 5B). The relatively high bootstrap value for each node supported the result.

Next, we isolated a contig encoding dopa decarboxylase (DDC), which catalyzes the final step in the synthesis of dopamine and serotonin. The *Lymnaea* DDC sequence (DDBJ accession number: FX190337) had a length of 1,763 bp and encoded 478 amino acids. Multiple alignments were performed and the result revealed highly conserved sequences of DDC among animal species (Fig. 6A). The phylogenetic tree with the neighbor-joining method showed that *Lymnaea* DDC, an only molluscan sequence in the tree, was distant from the DDCs of arthropoda and chordata species (Fig. 6B). The relatively high bootstrap value for each node further supported the notion that these were apparent taxa in this tree.

We further identified a sequence of *Lymnaea* tyramine beta hydroxylase (TBH), a homolog of dopamine beta hydroxylase (DBH), which catalyzes the last step in the synthesis of octopamine. The *Lymnaea* TBH sequence (DDBJ accession number: FX185559) had a length of 2,644 bp and encoded 584 amino acids. Protein sequences of other species TBH/DBH were identified by a blast search and the sequence alignment showed conserved positions among species (Fig. 7A). The amino acid sequence conservation of TBH was relatively low compared to the alignment results of TH and DDC (Figs. 5A and 6A), which was consistent with the previous report of DBH structural analysis [26]. A phylogenetic analysis clearly demonstrated that *Lymnaea* TBH of mollusca, as well as the freshwater planarian *Dugesia japonica* TBH of platyhelminthes, was distant from arthropoda and chordate TBH/DBH (Fig. 7B) and the relatively high bootstrap values also supported these results.

For these three sequences, we performed tblastx searches against GenBank, and confirmed that any nucleotide sequence in molluscan species has not been reported yet. Blastn searches against the public EST database were further performed and several molluscan sequences were found (7 sequences for DDC and 1 for TBH), but they did not code the whole ORFs. Therefore, the sequences were the first reported homologues of TH, DDC and TBH in molluscs. The cDNA sequences for *Lymnaea* TH, DDC and TBH were identified by RT-PCR experiments (Fig. S1 and Protocol S1) and the cloned sequences were compared to the TSA sequences. As a result, we found no, or very few, nucleotide differences between the cDNA and TSA sequences (Figs. S2 to S4). We further examined the read mapping with Bowtie [27] and confirmed that the positions of different nucleotide showed some mismatches between the contig and reads (data not shown). Thus, the nucleotide differences were possibly caused by single nucleotide polymorphisms (SNPs), or sequence specific errors (SSEs) as previously reported [28]. The obtained cDNA sequences have been deposited in DDBJ (accession numbers AB733115 to AB733117). These results further supported the accuracy of assembly in the present RNA-seq analysis.

## Discussion

In this study, we performed *de novo* transcriptome sequencing of the central nervous system (CNS) in *Lymnaea stagnalis* using an Illumina sequencer. First, we compared the five assembly programs, ABySS, Velvet, OASES, Trinity and Rnnotator, with respect to several well-known criteria, i.e., N50 length, maximum contig length and contig number (Figs. 1 and 2A). In addition, the coverage of known sequences was used as another criterion (Fig. 2B). The results showed that ABySS and Velvet were clearly inferior to OASES, Trinity and Rnnotator in the assembly of long transcriptome sequences. The large difference seems to have been caused by the different assembly strategies for transcriptome or genome analysis because transcriptome data includes a large amount of redundant sequence information, unlike genomic sequence data. ABySS and Velvet are the programs developed for *de novo* genome assembly with the de Bruijn graph method [14], [15]. In contrast, OASES, Trinity and Rnnotator were developed as *de novo* transcriptome assemblers with a strategy that merges the contigs obtained by the first assembly. It is also consistent with the previous report that Velvet itself is not sufficient for assembly of long contigs in *de novo* transcriptome sequencing with an Illumina Genome Analyzer [29].

Among OASES, Trinity and Rnnotator, we did not see a consistent difference in the criteria values (Fig. 2), and the only striking difference was the number of variants (OASES: 54,601; Trinity: 44,062; Rnnotator: 2,675). The datasets of OASES and Trinity included a large number of variants because they were designed to finely detect the variants as splicing products [16], [18]. Longer transcripts seemed to have a larger number of variants and longer variants as possible assemblies. In the comparison using the longest variants shown in Figure 2A, the reason of longer N50 and average contig lengths in OASES and Trinity can be considered to be the result of possible assembly. On the other hand, Rnnotator produced a small number of variants because of the error correction program at the post-processing step [19]. The effects of splicing variants or duplicate genes on Rnnotator data have not been studied yet. Even if the genetic variants are compressed into a small number of contigs, the redundant reads of variants are counted as the expression level with reads per kilo base per million (rpkm) values. Thus, we here selected the data of Rnnotator, including the smallest number of variants, for GO analysis. However, OASES and Trinity were also useful to obtain long transcripts. According to the purpose of research, these programs can be used singly or jointly as in the previous reports [24], [30].

The present results demonstrated that RNA-seq analysis using an Illumina sequencer was more efficient than the classical Sanger sequencing, in terms of both sequence quantity and quality, for *de novo* transcriptome sequencing. Rnnotator assembly produced 116,355 sequences as *Lymnaea* transcriptome shotgun assembly (TSA) and the data was compared to the previous *Lymnaea* expression sequence tag (EST) data (Table 3). The blast comparison demonstrated that about 5% of the present TSA data covered 80% of the previous EST, and about 110,000 sequences were proved to be newly obtained *Lymnaea* transcripts. The good assembly of the present RNA-seq analysis was also confirmed because a single contig of the present TSA was equivalent to several shorter sequences of the previous EST [8]. In GO analysis, the distributions of GO terms showed low similarity to the results of the previous study [8], and the large difference was caused by the different size of the two datasets.

The present study also demonstrated that the reported molluscan data are insufficient to cover the whole transcriptome data. The small number of molluscan sequences in the blast analyses (Table 2) and the phylum distribution of top hit species (Fig. 4) strongly supported this idea. These results did not indicate that *Lymnaea* sequences are more similar to the sequences of other phylum species, but rather that there is a lack of molluscan sequences in the public database. In addition, the present *Lymnaea* TSA showed low similarity to *Aplysia* EST (Table 4). A similar observation was made in a previous study, in which the EST obtained from juvenile *Aplysia* did not show similarity to the currently available *Aplysia* EST database [31]. Our findings, combined with the previous observations, suggest that the present data obtained by RNA-seq enlarge the molluscan transcriptome database substantially.

As representative examples of contig sequences, *Lymnaea* homologues of tyrosine hydroxylase (TH), dopamine decarboxylase (DDC) and tyramine beta hydroxylase (TBH) were selected (Figs. 5 to 7). We also confirmed the accuracy of their sequences by DNA sequencing of the cloned cDNA sequences (Figs. S1, S2, S3, S4). Those enzymes catalyze the synthesis of monoamines that play critical roles in various behavioural regulations [32]–[35]. The amino acid alignments showed that the predicted sequences of *Lymnaea* TH, DDC and TBH were reliable for conserved sequences among species. Phylogenetic analyses also revealed that the evolutional distances were reasonable because of the high boot strap values. The identification of these sequences further supported the accuracy of *de novo* transcriptome assembly in this study.

The development of techniques for RNA-seq has now enabled researchers to perform *de novo* sequencing of mollusc species. Recently, transcriptome sequencing in the pond snail *Radix balthica* [29] and genomic sequencing of the pearl oyster *Pinctada fucata* [36] were reported. The Illumina short read sequencer, when used together with a good assembly program, is highly useful, and its low cost-performance will speed up *de novo* sequencing. Further, the available genetic information in public databases will be valuable for future sequencing. Several studies have demonstrated that the genome of a closely related species can be used as a reference for *de novo* transcriptome assembly of non-model organisms [13], [37], [38]. In addition to raw sequencing data, the assembled sequences can now be updated in public databases, such as TSA in DDBJ and GenBank. Reliable data of good assembly sequences is continuously needed to enlarge the genetic database, not only for mollusc species but for all non-model species, as we demonstrated in this study.

### Conclusions

Using an Illumina short read sequencer and well developed assembly programs, we successfully performed *de novo* transcriptome sequencing. The assembly program Rnnotator produced 116,355 transcript sequences as a transcriptome shotgun assembly (TSA) in *Lymnaea* CNS. The present data improved the previous *Lymnaea* transcriptome data and further helped to enlarge the public database of mollusc species. Moreover, the present study using the RNA-seq method will facilitate the future genetic analyses of non-model animals.

## Supporting Information

Figure S1 Molecular cloning of *Lymnaea* TH, DDC and TBH cDNAs. A. RT-PCR was performed using primers that were designed according to the TSA sequences for *Lymnaea* TH, DDC and TBH. The ORFs are depicted as the colored boxes, and locations and directions of primers are shown by arrowheads. B. Electrophoresis gel showing RT-PCR products generated from cDNA sample of *Lymnaea* CNS. Used primer set with expected product length is shown for each lane. DNA ladder markers (Fermentas, Hanover, Germany) in the left and right lanes are labeled.
(PDF)

Figure S2 Sequence alignments of coding regions of the isolated cDNA and TSA for LymTH. The cDNA sequence is determined based on analysis of RT-PCR using a cDNA library sample derived from a single *Lymnaea* CNS. Nucleotide differences are shaded in black, and positions with codon differences are indicated by underlines when causing amino acid changes.
(PDF)

Figure S3 Sequence alignments of coding regions of the isolated cDNA and TSA for LymDDC. The cDNA sequence is determined based on analysis of RT-PCR using a cDNA library sample derived from a single *Lymnaea* CNS. One nucleotide difference without amino acid sequence changing was found.
(PDF)

Figure S4 Sequence alignments of coding regions of the isolated cDNA and TSA for LymTBH. The cDNA sequence is determined based on analysis of RT-PCR using a cDNA library sample derived from a single *Lymnaea* CNS. No nucleotide difference was found between LymTBH cDNA and TSA sequences.
(PDF)

Protocol S1 Method for molecular cloning of *Lymnaea* TH, DDC and TBH cDNAs.
(PDF)
