# Supplementary figures and images for: De Novo Sequencing and Transcriptome Analysis of the Central Nervous System of Mollusc Lymnaea stagnalis by Deep RNA Sequencing
Source: PLoS One. 2012 Aug 1;7(8):e42546. doi: 10.1371/journal.pone.0042546 (PMC3411651; doi:10.1371/journal.pone.0042546)

**A**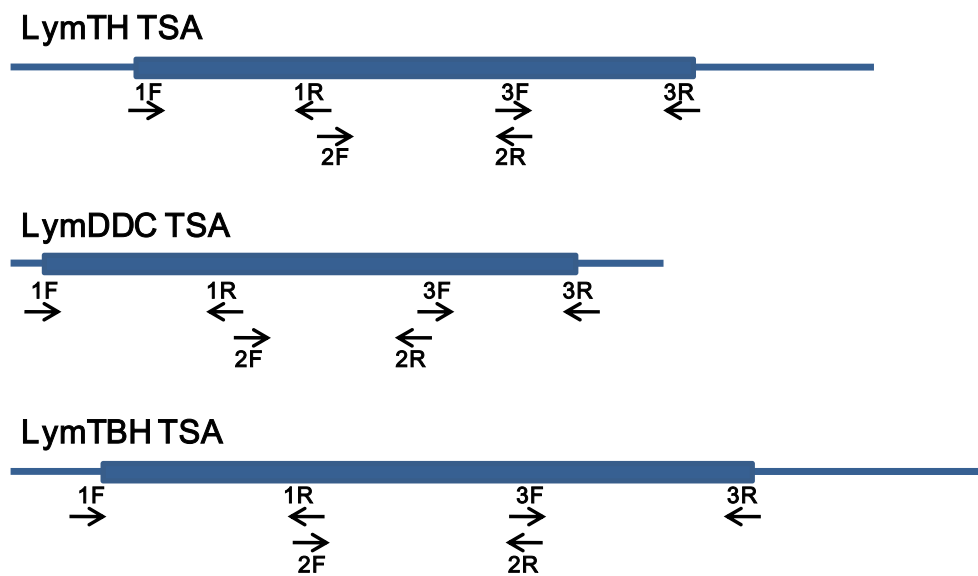**B**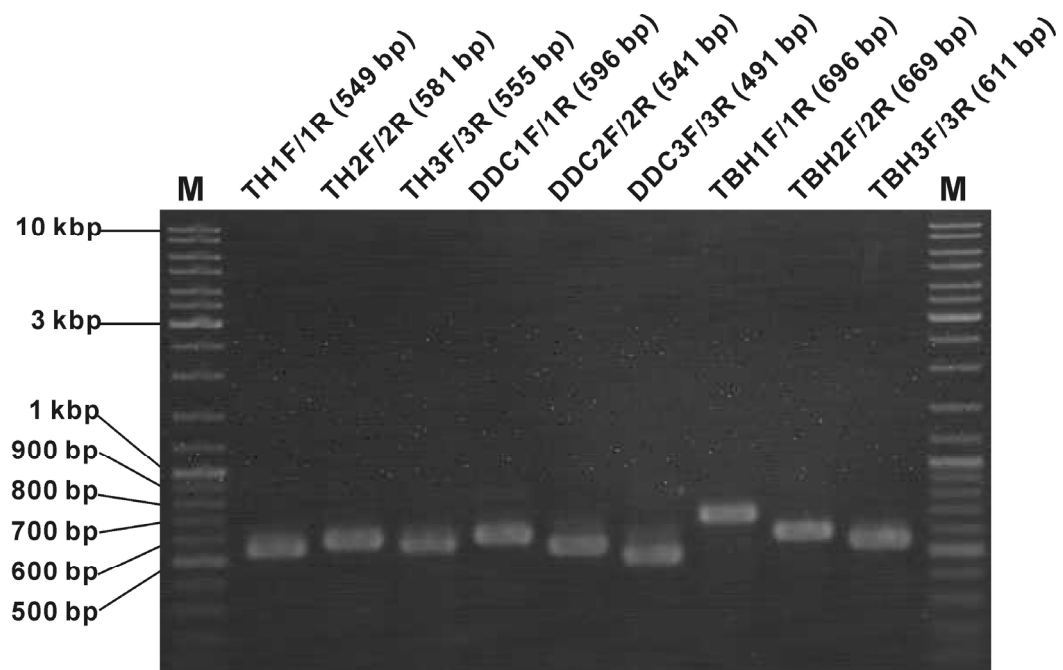

Supplement: Figure S1 — Molecular cloning of Lymnaea TH, DDC and TBH cDNAs. A. RT-PCR was performed using primers that were designed according to the TSA sequences for Lymnaea TH, DDC and TBH. The ORFs are depicted as the colored boxes, and locations and directions of primers are shown by arrowheads. B. Electrophoresis gel showing RT-PCR products generated from cDNA sample of Lymnaea CNS. Used primer set with expected product length is shown for each lane. DNA ladder markers (Fermentas, Hanover, Germany) in the left and right lanes are labeled. (PDF) [file pone.0042546.s001.pdf]
