# Supplementary material for: De Novo Sequencing and Transcriptome Analysis of the Central Nervous System of Mollusc Lymnaea stagnalis by Deep RNA Sequencing
Source: PLoS One. 2012 Aug 1;7(8):e42546. doi: 10.1371/journal.pone.0042546 (PMC3411651; doi:10.1371/journal.pone.0042546)
Supplement: Figure S2 — Sequence alignments of coding regions of the isolated cDNA and TSA for LymTH. The cDNA sequence is determined based on analysis of RT-PCR using a cDNA library sample derived from a single Lymnaea CNS. Nucleotide differences are shaded in black, and positions with codon differences are indicated by underlines when causing amino acid changes. (PDF) [file pone.0042546.s002.pdf]

LymTH

```
cDNA ATGAACGGGATTCCGGACGTCAACGAAACAGCCAAGCGACGGCTTGCATTTCAAAAAGCTTTCAGCCAAGAGCACGGTGG 80
TSA  ................................................................................ 80

     GTGGCGCAAGAAGAGCTTAATAGCAGACGCCAAGTTCGAGACAGCCGCCAACAAGGAGTTCAAGAAACAGGAGCGGCGCC 160
     ................................................................................ 160

     TCAGCAAAACAGACTCTGTCAGCGACGAGGAAGGATTCGCCCCTGGGCTTTTACCGGACGCCTTCCCGGAGGATTACGCC 240
     ................................................................................ 240

     GCCTTCAACCTCCTGGTGACTCTCAGGGAGGGAGCCATCAGCTTGGCCAAGGTGATCCGATGTTTTGAGAATGGCAAAGT 320
     ................................................................................ 320

     GTTCATCACCCACGTGGAGTCTCGTAAGGCGCTCTCAGATCTAAAGCAGTACCAGCTCTTCCTGCAGGTCGTCTGCACGC 400
     ................................................................................ 400

     ACGAGACGTTCGACAACGTCTGCGGTGCTGCCAGGCAAAGTCCACTCATTTCGGATCTCAAACTGTTGGAGGAAAAGGAG 480
     ................................................................................ 480

     CCAGAGAAAAAAGACATTTGGTTTCCCAAACACATCAGTGACCTGGACAAGTGTACACACCTCATTACAAGGTTTGAACC 560
     ................................................................................ 560

     AGATTTAGACTACACACATCCAGGTTTTGCTGACAAAAACTATAGATTGAGAAGAAAAGAAATCGCTGACATTGCCTTTG 640
     ..................................C.......C..................................... 640

     GTTACCGATGCGGACAAGCCATTCCCAGAGTGGAGTACACAGAAGAAGAGAACGCAACCTGGGCCCACGTGTATCGTCAT 720
     ..................................................G............................. 720

     CTCAAGGACCTGTTCCCGACCCACGCCTGCAAGGAGCACATCGACGTCTTCAAGTTGCTGGAGATGGAGGGCGGGTTTTG 800
     ..T............................................................................. 800

     CGAGGAGAAGATCCCCCAGCTGGAGGATGTCTCAAACTTTCTCAAACGTAAGACTGGGTTTCAGCTCCGTCCGGTGGCCG 880
     ........................A....................................................... 880

     GTCTACTCTCAGCCCGAGACTTCCTGGCATCCCTGGCGTTCAGAACCTTCCAGTGCACACAATACGTCAGGCACGGGGCC 960
     ................................................................................ 960

     AAGCCGGATCACTCGCCTGAGCCAGACTGCATCCACGAGTTACTCGGACACGTCCCCATGTTGGCTGACCCGAAGTTCGC 1040
     ................................................................................ 1040

     CCAGTTCGCCCAGGAGTTGGGGCTGGCCACTCTCGGTGTGTCGGACGAGAACATTGAGAAGTTCGCGACGTTATTTTGGT 1120
     ................................................................................ 1120

     TCACTGTTGAGTTCGGCCTCTGCAAACAAAACGGCGAGCTGAGGGCGTACGGGGCCGGCATGCTCTCCTCCTACGGGGAG 1200
     ................................................................................ 1200

     CTACAGAACAGCCTCAGTGGGGCGCCCACGGTGAAAGAGTTCGACCCTATGGTGACGGCGGTTCAGGAGTACAAGGACGA 1280
     ................................................................................ 1280

     CGACTTCCAGCCCATCCTGTTTGTCGTGGAGTCGTTTGAGGACATGATGACCAAGATGAGACAATACGTCGCCAGCATTG 1360
     ................................................................................ 1360

     ACCGAGAGTTCGACCTGAGTTACGACCCCTACACGCAGTCCGTCAAGATCCTGGACCACAATTCTGCACTGGAGGAGGTG 1440
     ................................................................................ 1440

     GCCGGTGGGCTGCAGCACGACGTCAACATGCTGGTCCACGTGATGAACAGGTTCAACAGGCCGGTCTAG            1509
     .....................................................................            1509
```
