# Supplementary material for: De Novo Sequencing and Transcriptome Analysis of the Central Nervous System of Mollusc Lymnaea stagnalis by Deep RNA Sequencing
Source: PLoS One. 2012 Aug 1;7(8):e42546. doi: 10.1371/journal.pone.0042546 (PMC3411651; doi:10.1371/journal.pone.0042546)
Supplement: Figure S3 — Sequence alignments of coding regions of the isolated cDNA and TSA for LymDDC. The cDNA sequence is determined based on analysis of RT-PCR using a cDNA library sample derived from a single Lymnaea CNS. One nucleotide difference without amino acid sequence changing was found. (PDF) [file pone.0042546.s003.pdf]

LymDDC

```
cDNA  ATGGACGCTCAAGAATTCCGTGCCAGGGGTCGCGAGATGGTCGACTACGTCGCTGACTACCTGGAGACCATCGGCACCCG 80
TSA   ................................................................................ 80

      CACTCCACTCCCTTCAGTCTTGCCTGGCTACCTGAGGGAGCTGATCCCAGACGAGGCGCCCCTGAATGGGGAGTCCTGGG 160
      ...........................G.................................................... 160

      AGGAGGTGAAAAAAGATATTGACAGGGTCATCATGCCAGGGGTCACCCACTGGCACAGCCCACAGTTCCATGCATACTTC 240
      ................................................................................ 240

      CCCACCTCCAGCTCTTACCCTGCCATTCTGGGGGACATGCTGTCTGACGGCATCGGGTGTATCGGATTCACGTGGCCAGC 320
      ................................................................................ 320

      AAGCCCCGCATGCACTGAGCTAGAAGTCTCCATGATGGACTGGTTGGCCAAGATGTTAAACCTGCCCCAAGAGTTTCTGT 400
      ................................................................................ 400

      TTAGTGGGGGAGGAAAAGGTGGAGGTGTTATACAGGGCACAGCAAGTGAGGCAACTCTTGTAGCACTCCTGTCGGCTAGG 480
      ................................................................................ 480

      ACCACCATGATTAATAAGCTCAAGAAAGACAACCCCCAAATGACCCAAGGGCAGATCGTTGATAAACTGGTGGCTTATTG 560
      ................................................................................ 560

      TTCAGAAGAGGCCCATTCATCAGTTGTTAGAGCCTCACTTATCGGCATGGTACAAATGAAATCTCTACCAACAGACGACA 640
      ................................................................................ 640

      AAGGATCCCTGAGAGGCTCGGAGCTAGAATCTGCCATCATTAAGGACAAGGAGCAAGGCTTGATACCTTTCTTTCTGTGT 720
      ................................................................................ 720

      GCAACTGTAGGGACCACATCCACATGTGGCACAGATAATTTGCTAGAACTTGGACCGATTTGTAACAAACATGACATCTG 800
      ................................................................................ 800

      GATGCATGTTGACGCCGCCTATGCCGGAAGTGCTTTCATATGTCCTGAGTTCAGACCTCTGCTGGATGGTGTGGAGCATT 880
      ................................................................................ 880

      CCATGTCTTTCAATTTCAACCCCCACAAATGGCTGCAGGTGACCTTTGACTGTTCAGCATTGTGGGTCAAGGACAGTGGT 960
      ................................................................................ 960

      CTGGTATCTGGGGCGTTTGAGTTGAACCCCGTTTACCTTAATCATGACAACCAGGGACAAGCCATGCCCGACTATAGACA 1040
      ................................................................................ 1040

      CTGGCAGATTCCGTTGGGCAGGAGGTTCCGGTCTCTAAAACTCTGGTTTGTTCTACGCATGTTTGGCGTCACAGGACTCC 1120
      ................................................................................ 1120

      AGGAACAAATAAGGAAGGATGTATCCCTTGCTCATCAGTTTGAGGACCTAGTTAAGAGTGATGACAGGTTTGAAATAGTC 1200
      ................................................................................ 1200

      AGAAAGGTGACCTTTGGCCTTGTTTGCTTTAGGTTAAAGGGGACCAATGAAATCAATGAAACACTTACAAAGAAAATAAA 1280
      ................................................................................ 1280

      CGATGACCGAAGGATCCACCTGACCCCATCCAAGGTCAAGGACACATTCTTTTTACGATTTGCCGTGTGCGCCACAAAGA 1360
      ................................................................................ 1360

      CCCAGGTTTCTGACGTTAAATTTGCTTGGACGGTTATACAAGAGCTGACCGACAGTTTGCTGTCCAGTCCAAAGTAG    1437
      .............................................................................    1437
```
