# Supplementary material for: De Novo Sequencing and Transcriptome Analysis of the Central Nervous System of Mollusc Lymnaea stagnalis by Deep RNA Sequencing
Source: PLoS One. 2012 Aug 1;7(8):e42546. doi: 10.1371/journal.pone.0042546 (PMC3411651; doi:10.1371/journal.pone.0042546)
Supplement: Figure S4 — Sequence alignments of coding regions of the isolated cDNA and TSA for LymTBH. The cDNA sequence is determined based on analysis of RT-PCR using a cDNA library sample derived from a single Lymnaea CNS. No nucleotide difference was found between LymTBH cDNA and TSA sequences. (PDF) [file pone.0042546.s004.pdf]

LymTBH

```
cDNA ATGGATGGACACGCTTGGTCGATCTCGGCTGTCGTCTGGCTCGTGGTTCTCTCGGCTGACCACTCGGTTCAAGCGTATCA 80
TSA  ................................................................................ 80

     GTATCAAGTGACCCTAGACCCCGACAGCAGGTACCTGTTCCAGTGGTCAGTGGACTACAAGGAAAGTCTTATCAATGTCC 160
     ................................................................................ 160

     AGCTCACGTGCAAGGTCACGCCAGAGAGCTGGCTTGCTTTTGGATTCTCGGACTACGGTGACGTCACTTCCGCTGACCTA 240
     ................................................................................ 240

     ATCTTGTTTTGGACAGACGGTGATGGCAAGCACCATTTCTTCGATGGTCACACGACACCGGATGGGATATTTCTACCTGA 320
     ................................................................................ 320

     CCGGCAACAGGACTACCATCTGACATCAGTCGCCGATGACCGTGGGTCAGTTGTGCTGGATTTCTACAGGCACTTCAACA 400
     ................................................................................ 400

     CATGCGACCCAGAGGACTACGCCCTGGACAACGGCACCACGCATCTCGTGTACGTAGAGTCGGCGCAGCCCGAGGGCCCG 480
     ................................................................................ 480

     CCCCTCGCCCGTGACGTCACGCGACTACGTCACGGGGTCCAACGCCTGCAGCTGTTAAAACCCGAGATTTCGGCCCCAGT 560
     ................................................................................ 560

     GTTCCCCGAGGACACGTGGTCGTTTGAGGTCAGGGCGCCAGAGGTGTTGGTGCCAGCCGAGGAGACGACCTACTGGTGGC 640
     ................................................................................ 640

     ACACTACCATCTTACCGGACATGCCATCACCCCACCACATCATACAGTACGAGGGGATCGTGGCGGAGGGAAGCGGTGAC 720
     ................................................................................ 720

     CTCGTCCACCACATGGAAGTCTTCCACTGTCAGGTCCAAAAAGGTCACGGCGTCCCGTACTACAATGGGCCCGGCATAGC 800
     ................................................................................ 800

     TGAAGGGAAGCCCGAAGGCCTGGAGGTGTGCAGGAAGGTCATCGGCGCGTGGGCCATGGGGGCGGAGGCGATGATATACC 880
     ................................................................................ 880

     CCGAGGAGGCAGGGGTCCCCGTGGGAGGCCAGGGGTTCTCGCGCTTTGCCTTGCTTGAGGTCCACTACAACAACCCACAG 960
     ................................................................................ 960

     AAGAAATCTGGTCGAATGGACTCGTCCGGGATCCGCTTCCACGTGACGTCACAACTCCGGAAGTATGACGCGGGGATCAT 1040
     ................................................................................ 1040

     GGAGCTCGGACTGGAGTACGTCAACAAGATGGCCGTGCCGCCCGGACAGCGGGACTTCAAGCTGAGCGGTTACTGCGTAC 1120
     ................................................................................ 1120

     ACAAATGTACACAAATGAGCTTACCACCTGCCGGCATCCACGTGTTCGCCTCCCAGCTCCACACTCACCTGACGGGGCGC 1200
     ................................................................................ 1200

     CGCGTGTACACCAAGCACGCGCGGGATGGCGCGGAGCTTCCGGAAGTCAACCGGGACAACCACTACAGCCCCCACTTCCA 1280
     ................................................................................ 1280

     GGAGATCAGGAGGCTGCCGCAGCCGCACCACGTCCTCCCGGGCGACGTGCTGATAACCACGTGCGAGTACGACACAACGA 1360
     ................................................................................ 1360

     AGAGGTCAAAGGCTACAGTTGGCGGCTTCTCCATAACAGATGAGATGTGTCTCAACTACGTCCACTACTACCCCAGGTCT 1440
     ................................................................................ 1440

     GACCTTGAAGTCTGCAAGAGCTCAGTCAGGACTGATTCGCTTCACACATTTTTCTTTTTGCTCAACAGATTCGAGAATTC 1520
     ................................................................................ 1520

     AAAGGTGTCTCCAGAGGCGGGTGACAGGGCAAACTATGAAAGCATTGAGTGGTCTCCCCTGAATGTCCGGCTCCTGGAGG 1600
     ................................................................................ 1600

     ATCTGTACAGCACCTCGCCGCTCTCCATGCAGTGCAACAGGTCAGACGGCACCCGGTTCCCCGGCGAGTGGGAGCACGTT 1680
     ................................................................................ 1680

     CGAGTCCCAGACATTGTCCGACCGTTGGTTGTGGACACGTCAGAAGTTTGCTCGGGCCACGTCACAGCGGAATAG      1755
     ...........................................................................      1755
```
