# Supplementary material for: De Novo Sequencing and Transcriptome Analysis of the Central Nervous System of Mollusc Lymnaea stagnalis by Deep RNA Sequencing
Source: PLoS One. 2012 Aug 1;7(8):e42546. doi: 10.1371/journal.pone.0042546 (PMC3411651; doi:10.1371/journal.pone.0042546)
Supplement: Protocol S1 — Method for molecular cloning of Lymnaea TH, DDC and TBH cDNAs. (PDF) [file pone.0042546.s005.pdf]

Protocol S1

RNA was extracted from dissected one *Lymnaea* CNS using an RNAqueous-Micro kit (Applied Biosystems/Ambion, Foster City, CA) and treated with DNase I (Ambion). Total RNA was used for the reverse transcription reaction with M-MLV Reverse Transcriptase (Invitrogen, Carlsbad, CA) and random primers according to the manufacturer's instructions. The RT products were applied to PCR experiments using ExTaq DNA polymerase (Takara Co., Osaka, Japan). To obtain cDNA fragments of *Lymnaea* tyrosine-hydroxylase (TH), dopa decarboxylase (DDC) and tyramine beta hydroxylase (TBH), PCR amplification was performed. Sequence-specific primers were designed according to the Rnnotator assembled *Lymnaea* sequences for TH (DDBJ accession number: FX186872), DDC (DDBJ accession number: FX190337) and TBH (DDBJ accession number: FX185559). The sequences of the designed primers were as follows: The primer sequences were as follows: TH1F, 5'-CGA ACT CTT TCA TCC ACC ATG AAC GGG A-3'; TH1R, 5'-CTT GTC CAG GTC ACT GAT GTG TTT GGG-3'; TH2F, 5'-CAT TTG GTT TCC CAA ACA CAT CAG TGA CCT-3'; TH2R, 5'-CGA GAG TGG CCA GCC CCA ACT-3'; TH3F, 5'-TGA GCC AGA CTG CAT CCA CGA GTT-3'; TH3R, 5'-CGC ATC TCT GTA GTG ACC CCT TCC TAG A-3'; DDC1F, 5'-GGT TGC ACC GGA CAC TAG CGT TTT-3'; DDC1R, 5'-GTT TAT CAA CGA TCT GCC CTT GGG TCA TT-3'; DDC2F, 5'-CCA AAT GAC CCA AGG GCA GAT CGT TG-3'; DDC2R, 5'-CAA CGG AAT CTG CCA GTG TCT ATA GTC GG-3'; DDC3F, 5'-AGG GAC AAG CCA TGC CCG ACT-3'; DDC3R, 5'-AGA TTT CAA TGG CCC ACA TCG CTT TCC-3'; TBH1F, 5'-GAA GTT GTT ACC GCA GGG CAT ATT ATA CAC-3'; TBH1R, 5'-TCT GGC GCC CTG ACC TCA AAC-3'; TBH2F, 5'-TCC AAC GCC TGC AGC TGT TAA AAC C-3'; TBH2R, 5'-GAG TGT GGA GCT GGG AGG CGA A-3'; TBH3F, 5'-TCG CCT CCC AGC TCC ACA CTC-3'; TBH3R, 5'-CCT TAC GCC GTC GCA TGG TTC TAT TC-3'. The amplified PCR fragments were subcloned into TOPO™ vector (Invitrogen), and at least three clones for each sample were subjected to nucleotide sequence analysis.
